# Supplementary material for: Designing antifilarial drug trials using clinical trial simulators
Source: Nat Commun. 2020 Jun 1;11:2685. doi: 10.1038/s41467-020-16442-y (PMC7264235; doi:10.1038/s41467-020-16442-y)
Supplement: Supplementary file 1 — Supplementary Information [file 41467_2020_16442_MOESM1_ESM.pdf]

Supplementary Information

Designing antifilarial drug trials using clinical trial simulators

Martin Walker[1,2,*], Jonathan I. D. Hamley[2,3,†], Philip Milton[2,3,†], Frédéric Monnot[4], Belén Pedrique[3], Maria-Gloria Basáñez[2,3]

[1] London Centre for Neglected Tropical Disease Research, Department of Pathobiology and Population Sciences, Royal Veterinary College, University of London, Hertfordshire, AL9 7TA, UK

[2] London Centre for Neglected Tropical Disease Research, Department of Infectious Disease Epidemiology, Imperial College London, London, W2 1PG, UK

[3] MRC Centre for Global Infectious Disease Analysis, Department of Infectious Disease Epidemiology, Imperial College London, London, W2 1PG, UK

[4] Drugs for Neglected Diseases initiative, 15 Chemin Louis-Dunant 1202 Geneva, Switzerland

* corresponding author mwalker@rvc.ac.uk
† contributed equally

Table of Contents

List of Figures **3**

List of Tables **4**

Supplementary Notes **5**

Simulations for an upper-end mesoendemic setting **5**

Treatment response dynamics 5

Opportune follow-up timeframes 5

Estimated sample sizes 5

Supplementary Methods **10**

EPIONCHO-IBM **10**

Human demography 11

Exposure to blackfly bites 11

Human host infection 13

Parasite demography 14

Onchocerca volvulus larvae and vector population 17

Single treatment with ivermectin 19

Treatment with a hypothetical macrofilaricide 20

Infection intensity and prevalence measures 20

Clinical trial outcome measures **22**

Sample size calculations **23**

Supplementary References **27**

List of Figures

Supplementary Figure 1. Parasitological response dynamics in ivermectin-treated and macrofilaricide-treated participants. 7

Supplementary Figure 2. Differences in parasitological outcomes between ivermectin-treated and macrofilaricide-treated participants 8

Supplementary Figure 3. Sample sizes required to detect a superior response in macrofilaricide-treated compared to ivermectin-treated participants. 9

Supplementary Figure 4. The relationship between the expected number of individuals eligible for participation in a clinical trial with increasing microfilarial intensity inclusion criterion........ 23

Supplementary Figure 5. Distribution of Welch's *t*-statistic generated from 1,000 clinical trial simulations using an inclusion criterion of > 0 microfilariae per mg. 24

Supplementary Figure 6. Distribution of Welch's *t*-statistic generated from 1,000 clinical trial simulations using an inclusion criterion of > 4 microfilariae per mg 25

Supplementary Figure 7 Distribution of Welch's *t*-statistic generated from 1,000 clinical trial simulations using an inclusion criterion of > 8 microfilariae per mg. 26

List of Tables

Supplementary Table 1. EPIONCHO-IBM state variable definitions 10

Supplementary Table 2. EPIONCHO-IBM parameter definitions for human host demography....... 11

Supplementary Table 3. EPIONCHO-IBM parameter definitions for exposure to blackfly bites...... 12

Supplementary Table 4. EPIONCHO-IBM parameter definitions for human host infection............ 13

Supplementary Table 5. EPIONCHO-IBM parameter definitions for parasite demography............ 16

Supplementary Table 6. EPIONCHO-IBM parameter definitions for larval stages within the vector and adult female blackfly population dynamics........ 18

Supplementary Table 7. EPIONCHO-IBM parameter definitions for the anti-parasitic effects of a single treatment with ivermectin......... 19

Supplementary Table 8. EPIONCHO-IBM parameter definitions for the anti-parasitic effects of treatment with a hypothetical macrofilaricide......... 20

Supplementary Table 9. EPIONCHO-IBM parameter definitions for infection intensity and prevalence measures......... 21

Supplementary Notes

Simulations for an upper-end mesoendemic setting

Treatment response dynamics

The treatment response dynamics in the mean number of mf/mg of skin (microfilarial intensity) and the percentage of participants positive for mf (microfilarial prevalence) in an upper-end mesoendemic setting (50% microfilarial prevalence among individuals ≥ 5 years) are illustrated in Supplementary **Error! Reference source not found.**. These dynamics are qualitatively very similar to the treatment dynamics in a lower-end mesoendemic setting (with a pre-treatment microfilarial prevalence of 40%; see Figure 3 main text) albeit the microfilarial intensity in participants is marginally greater in the upper-end mesoendemic setting because of the increased intensity of transmission (increased annual biting rate; 550 compared to 400 bites per person per year).

Opportune follow-up timeframes

The differences between microfilarial intensity and prevalence outcome measures among participants treated with either ivermectin or a macrofilaricide (a macrofilaricidal-only macrofilaricide, MOM or a macrofilaricidal and microfilaricidal macrofilaricide, MAMM) with different assumed efficacies are shown in Supplementary Supplementary Figure **2**. Analogous to the simulations conducted for a lower-end mesoendemic setting (see Figure 4 in the main text), at follow-up times less than 12 months after treatment, ivermectin is observed to be either superior or equally efficacious as the macrofilaricides (MOM/MAMM; because of its rapid microfilaricidal activity). The MOM begins to elicit a superior response compared to ivermectin between 12 and 24 months after treatment and the MAMM yields superior reductions in microfilarial load compared to ivermectin sooner, between 6 and 12 months (depending on macrofilaricidal efficacy). The principal difference between Supplementary Supplementary Figure **2** and Figure 4 (main text) is the noticeably increased uncertainty in the difference between the (average) microfilarial intensity between ivermectin- and macrofilaricide-treated participants (compare Supplementary Figure 2a and 2b with Figure 4a and 4b in the main text).

Estimated sample sizes

Estimated sample sizes required to detect with 80% power a statistically significant superior response (using either microfilarial intensity or prevalence) in the macrofilaricide-treated test group compared to the ivermectin-treated control group in an upper-end mesoendemic setting at various times after treatment, macrofilaricidal efficacies, inclusion criteria and number of skin snips are shown in Supplementary Supplementary Figure **3**. These estimates are analogous to those presented in Figure 5 of the main text for a lower-end mesoendemic setting. Qualitatively, the patterns shown in

Supplementary Supplementary Figure **3** and Figure 5 are very similar. Sample sizes decrease with increasing macrofilaricidal efficacy, marginally for microfilarial intensity (because of the increased measurement precision) for 4 versus 2 skin snips and when using microfilarial prevalence compared to microfilarial intensity with an inclusion criterion of > 0 mf/mg. Moreover, Supplementary Supplementary Figure **3** shows more explicitly than Figure 5 the opposing effects on sample sizes for the different outcome measures of increasing the minimum microfilarial intensity inclusion criterion. Namely, increasing the inclusion criterion decreases required sample sizes for microfilarial intensity but increases sample sizes for microfilarial prevalence.

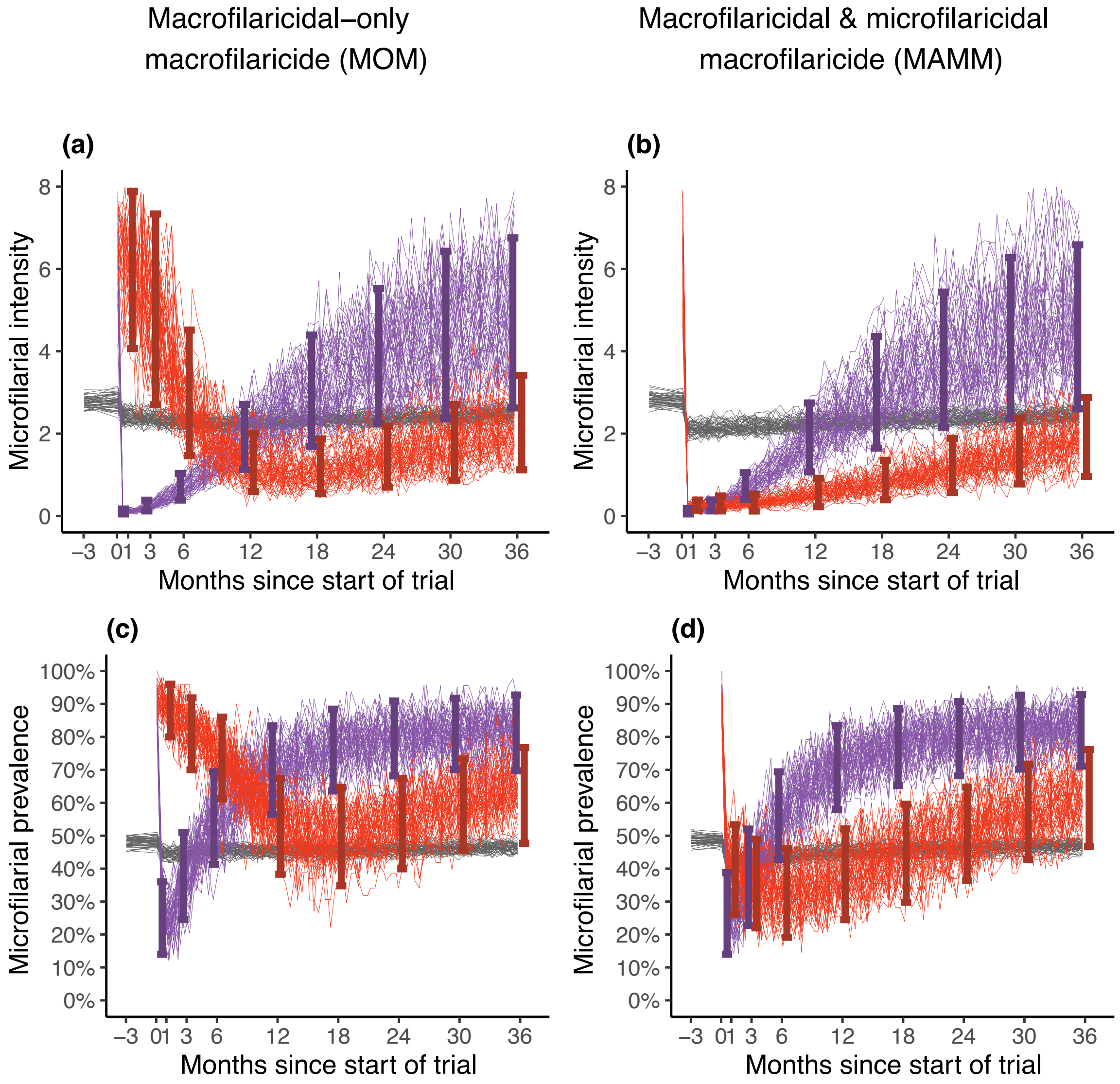

Supplementary Figure 1. Parasitological response dynamics in ivermectin-treated and macrofilaricide-treated participants.

Each panel depicts parasitological response dynamics in two 50-participant cohorts treated with either ivermectin (purple lines) or a hypothetical macrofilaricide (red lines) in an ivermectin-naïve upper-end mesoendemic setting. The macrofilaricide has an efficacy of 90% (90% of adult *Onchocerca volvulus* worms are killed within three months of treatment) and either macrofilaricidal activity only (MOM, left-hand side; panels (a) and (c)) or macrofilaricidal and microfilaricidal activity (MAMM, right-hand side; panels (b) and (d)). The parasitological outcome measure is either the arithmetic mean number of microfilariae (mf) per mg of skin (microfilarial intensity, panels (a) and (b)) or the percentage of participants positive for mf (microfilarial prevalence, panels (c) and (d)), both measured by 2 skin snips. Participants were included in the cohort if they were positive for mf (i.e. inclusion criterion was > 0 mf/mg of skin). Each thin line represents a single simulation and the vertical error bars indicate the range which captures 95% of the simulations. The grey lines indicate the mean (either mf per mg of skin, i.e. intensity, or presence of mf, i.e. prevalence) in the whole population, which is assumed to comprise 1,000 individuals (from which trial participants would have been recruited). Note that only eligible participants are treated, i.e. (community-wide) mass drug administration is not simulated.

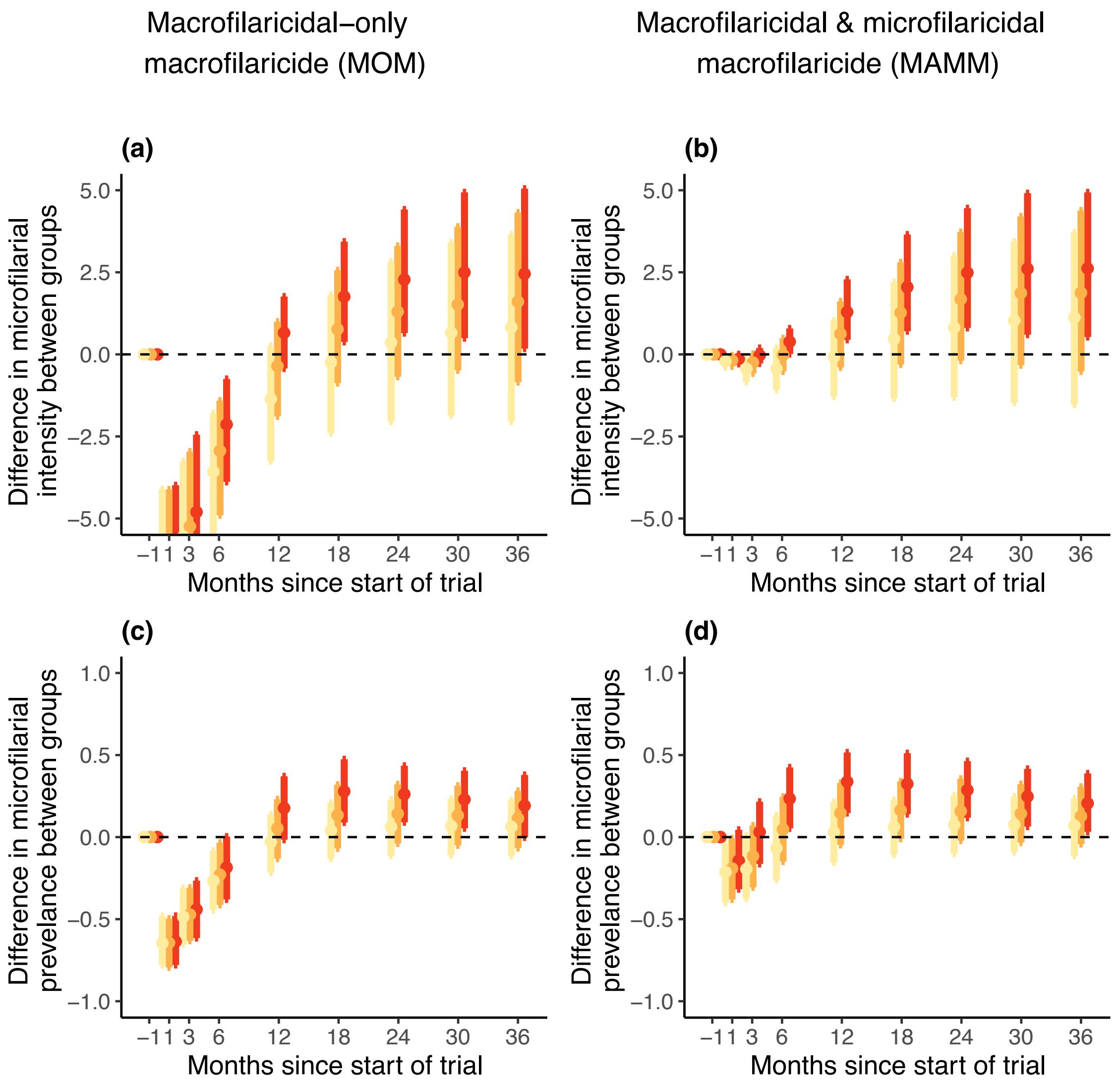

Supplementary Figure 2. Differences in parasitological outcomes between ivermectin-treated and macrofilaricide-treated participants

Each panel depicts differences in parasitological outcome measures between two 50-participant cohorts treated with either ivermectin (control group) or a hypothetical microfilaricide (test group) in an ivermectin-naïve upper-end mesoendemic setting. The macrofilaricide has an assumed efficacy of either 60% (cream points and lines), 75% (light orange points and lines) or 90% (red points and lines). Efficacy refers to the percentage of adult *Onchocerca volvulus* killed within three months of treatment. Left-hand side panels (a) and (c) show results for a macrofilaricidal only macrofilaricide (MOM); right-hand side panels (b) and (d) show results for a macrofilaricidal and microfilaricidal macrofilaricide (MAMM). The parasitological outcome measures compared are either the difference in arithmetic mean number of microfilariae (mf) per mg of skin (microfilarial intensity, panels (a) and (b)) or the difference in percentage of participants positive for mf (microfilarial prevalence, panels (c) and (d)), both measured by 2 skin snips. Participants were included in the cohort if they were positive for mf (i.e. infection status inclusion criterion was > 0 mf/mg of skin). Points indicate the arithmetic mean of the simulated differences in the average response between treatment groups and vertical error bars indicate the range which captures 95% of the differences. The horizontal dashed line indicates the threshold of equivalence (i.e. difference = 0), above or below which the MOM/MAMM or ivermectin exhibit superiority, respectively.

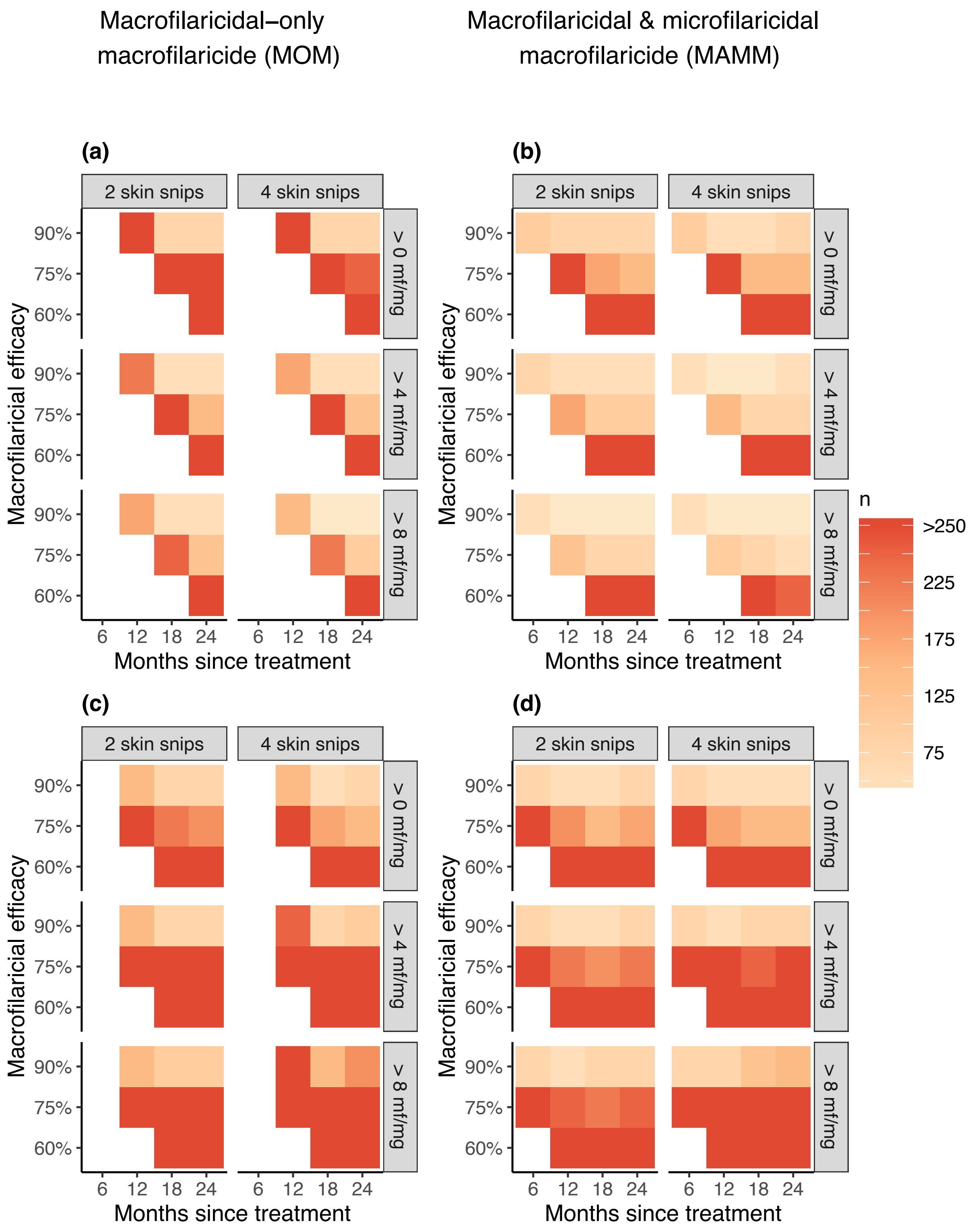

Supplementary Figure 3. Sample sizes required to detect a superior response in macrofilaricide-treated compared to ivermectin-treated participants.

Each panel depicts sample sizes (*n*) required to detect, with 80% power, a statistically significant superior response in the macrofilaricide-treated test group compared to the ivermectin-treated control group in an ivermectin-naïve upper-end mesoendemic setting. Sample sizes are calculated at 6, 12, 18 and 24 months after treatment assuming a 10% loss to follow up per year. Macrofilaricidal

efficacy (either 60%, 75% or 90%) corresponds to the percentage of adult *Onchocerca volvulus* killed within three months of treatment with a macrofilaricidal only macrofilaricide (MOM, left-hand side; panels (a) and (c)) or a macrofilaricidal and microfilaricidal macrofilaricide (MAMM, right-hand side; panels (b) and (d)). The parasitological outcome measures are the arithmetic mean number of microfilariae (mf) per mg of skin (microfilarial intensity, panels (a) and (b)) and the percentage of participants positive for mf (microfilaria-positive, panels (c) and (d)), both measured by 2 or 4 skin snips and using an infection eligibility criterion before treatment of either > 0 (i.e. presence of mf), > 4 or > 8 mf/mg of skin. Empty squares correspond to time points when the response in the macrofilaricidal-treated test group is inferior to ivermectin.

Supplementary Methods

EPIONCHO-IBM

Here we describe the stochastic, individual-based analogue of the EPIONCHO transmission model parameterised for onchocerciasis transmission and control in African savannah epidemiological and entomological context.[1,2] Human stages of infection are modelled for each individual human host in the population using stochastic difference equations (for adult *Onchocerca volvulus*) and deterministically using partial differential equations (for microfilariae). Vector stages of infection (L1, L2 and L3 larvae) are modelled deterministically using ordinary differential equations. Changes in the worm populations within an individual human are defined by stochastic transition events, in which the number of events is drawn from a binomial distribution with the probability of a transition being the product of the event rate and the (small) time step, dt. We introduce and define the notation for parasite state variables using the following subscripts: subscript a is context dependent, denoting the age of humans or parasites; subscript s is also context dependent, denoting the sex of humans or of adult worms; subscript l (life stage) is used for rates in parasite life history and is either W (adult *O. volvulus*) or M (microfilariae); subscript i indicates quantities that are specific to an individual human host, such as their age, sex or relative exposure to blackfly bites. Supplementary Supplementary Table **1** provides the definitions of state variables in EPIONCHO-IBM.

Supplementary Table 1. EPIONCHO-IBM state variable definitions

State variable	Definition
$W_{s(i,a)}$	number of adult *Onchocerca volvulus* of sex s ($s = F$ or $s = M$) aged a in human host i
$W_{FN(i,a)}$	number of non-fertile adult female *O. volvulus* aged a in human host i
$W_{FF(i,a)}$	number of fertile adult female *O. volvulus* aged a in human host i
$M_{(i,a)}$	number of *O. volvulus* microfilariae aged a in human host i
$L1$	mean number of (sausage) L1 *O. volvulus* larvae per blackfly vector

$L2$	mean number of (pre-infective) L2 *O. volvulus* larvae per blackfly vector
$L3$	mean number of (infective) L3 *O. volvulus* larvae per blackfly vector

Human demography

The human population (subscript H) is closed, with all deaths matching births to maintain a constant population size, N_H. The human mortality rate is assumed to be constant with age, such that the probability of survival at age a, is given by,

$$S_H = e^{-a\mu_H}. \quad (1)$$

It follows that the number of deaths at each time step is a random variable drawn from a binomial distribution, $B(N_H, \mu_H dt)$. Exponential distributions are unbounded for positive values and thus the distribution is truncated to prevent excessively long lifespans; a maximum age, $a_{\max} = 80$, is implemented, at which all individuals die. Newborns are uninfected (i.e. there is no vertical transmission of parasites, although *in utero* transmission of microfilariae has been reported).[3] Individuals are assigned a sex $s = F$ or $s = M$ randomly, with an equal probability of assignment, $\psi_F = \psi_M = 0.5$. (Note that we later also use subscript s to denote the sex of adult parasites and hence the definition of s is context dependent.) Women aged between 16 and 40 years become pregnant at a per capita rate r (pregnancy excludes women from antifilarial treatment). Once pregnant, it is assumed that women do not get pregnant again for a period of 2 years, representing both gestation and post-natal breastfeeding. When $r = 0.05$, approximately 10% of women between 16 and 40 years old are pregnant or breastfeeding at any time, with the maximum proportion of women pregnant or breastfeeding occurring in the 35 to 40 age group (Figure 2b, main text). Supplementary Supplementary Table **2** provides the definitions for human host demography.

Supplementary Table 2. EPIONCHO-IBM parameter definitions for human host demography

Parameter	Definition	Value	Reference
N_H	number of human hosts modelled in population	1,000	this paper
μ_H	per capita mortality rate of human hosts	0.02 year^{-1}	Basáñez & Boussinesq[4]
a^{max}	maximum age of human hosts	80 years	Filipe et al.[5]
ψ_s	probability that a human host is of sex s	$\psi_F = \psi_M = 0.5$	Filipe et al.[5]
r	per capita rate at which women aged between 16 and 40 years become pregnant	0.05 year^{-1}	this paper

Exposure to blackfly bites

Individuals within the population are differentially exposed to blackfly bites dependent on their age, sex, and an individual-specific exposure, $E_{(i)}$ (i.e. human hosts are differentially exposed to blackfly

bites). This individual-specific exposure factor is assigned at birth and is drawn form a gamma distribution,

$$E_{(i)} \sim G(k_E, \beta_E), \tag{2}$$

where k_E and β_E are the shape and rate parameters respectively. We assume that $k_E = \beta_E$ such that the mean exposure in the population is unity. This ensures that blackfly bites are distributed among hosts with an average exposure that is given by the annual biting rate (number of bites per person per year) and that the distribution of adult worms is overdispersed, characteristic of helminth parasites (Figure 2c, main text).

Exposure to blackfly bites changes with individual host age and is different for men and women. Following Filipe et al.,[5]

$$\Omega_s(a_{(i)}) = E_s \cdot \gamma_s \cdot E_0 \qquad \text{for } a_{(i)} < q$$

$$\Omega_s(a_{(i)}) = E_s \cdot \gamma_s \cdot exp^{[-\alpha_s(a_{(i)}-q)]} \qquad \text{for } a_{(i)} \geq q \tag{3}$$

where E_s is the sex-specific exposure to vector bites (calculated from the relative exposure of males versus females $Q = E_M/E_F$); E_0 is the fraction of exposure at age 0 relative to the age from which exposure changes continuously with age (when $a_{(i)} \geq q$), and α_s is the sex-specific change in contact rate between vectors and hosts with increasing host age. This is multiplied by the normalization factor γ_s to ensure that the distribution of bites among age groups sums to 1. An individual's (relative) age- and sex-specific exposure is combined with their individual-specific biting factor to produce their total exposure to blackfly bites,

$$\Omega_T(a_{(i)}) = E_{(i)}\Omega_s\,(a_{(i)}). \tag{4}$$

Supplementary Supplementary Table **2** provides the definitions for human exposure to vector bites.

Supplementary Table 3. EPIONCHO-IBM parameter definitions for exposure to blackfly bites

Parameter	Definition	Value	Reference
k_E, β_E	shape and rate parameters of the gamma distribution describing individual human hosts exposure to blackfly bites	$k_E = \beta_E = 0.3$	Hamley et al.[6]
$Q = E_M/E_F$	relative male to female exposure to blackfly bites	1.20	Filipe et al.[5]
α_F	rate of change in exposure to blackly vectors with age among females	-0.023 year^{-1}	Filipe et al.[5]
α_M	rate of change in exposure to blackly vectors with age among males	0.007 year^{-1}	Filipe et al.[5]
q	period (age) preceding initial increase in exposure to vector bites during childhood	0 years	Filipe et al.[5]

Human host infection

Human host infection dynamics are modelled by stochastic and deterministic simulation. The rate at which infective L3 larvae in vectors are acquired by the host is governed by the annual transmission potential (ATP), calculated as the number of L3 larvae received per person per year. The ATP at time t is the product of the per blackfly biting rate on humans, $\beta = h/g$ (where h is the human blood index, the fraction of bloodmeals taken on humans and g is the duration of the gonotrophic cycle taken as the period between two consecutive bloodmeals and assuming gonotrophic concordance, i.e. one bloodmeal leads to the production of one batch of eggs), the ratio of vectors to hosts, V/H, and the temporally dynamic mean number of infective L3 larvae per blackfly vector, $ATP(t) = \beta(V/H)L3(t)$. The quantity V/H is inferred from the annual biting rate $ABR = \beta\, V/H$ which is the key input which adjusts the endemicity level of EPIONCHO-IBM simulations. Establishment of larvae in human hosts is negatively density (transmission intensity) dependent, with the per capita rate of worm establishment decreasing with increasing ATP.[7] This density dependency is controlled by the function $\Pi_H[ATP(t)]$ representing the (decreasing) proportion of L3 larvae developing to adult worms within the human host as a function of (increasing) ATP. We assume a delay τ_H between L3 larvae entering the host and establishing as adult worms to account for the duration of development into L4 larval stages and L5 (juvenile adults). Thus, individual acquisition of male and female adult worms (where sex is denoted by subscript $s = F$ or $s = M$) is modelled as a discrete-time stochastic process,

$$W_{s(i,1)}(t) \rightarrow W_{s(i,1)}(t + dt) + 1 \qquad (5)$$

with probability: $\psi_s \cdot \Omega_T(a_{(i)} - \tau_H)ATP(t - \tau_H)\Pi_{H_{(i)}}\left[ATP(t - \tau_H), \Omega_T(a_{(i)} - \tau_H)\right] dt$

where $W_{s(i,1)}$ is the number adult worms of sex s in the first worm age group and ψ_s is the (equal) probability that an incoming parasite is male or female, such that $\psi_M = \psi_F = 0.5$. Supplementary Supplementary Table **4** provides the parameter definitions for human host infection.

Supplementary Table 4. EPIONCHO-IBM parameter definitions for human host infection

Parameter	Definition	Value	Reference
$\beta = h/g$	per blackfly biting rate on humans, calculated as the product of the proportion of blackfly bites taken on humans (the human blood index) and the reciprocal of the duration of the gonotrophic cycle	$h = 0.63$<br>$g = 1/104$ year^{-1}	Lamberton et al.[8]<br>Basáñez and Boussinesq[4]

$ABR = \beta V/H$	annual biting rate of blackflies on humans; the key variable for simulating different endemicity levels	400 $year^{-1}$ (lower-end mesoendemicity)<br>550 $year^{-1}$ (upper-end mesoendemicity)	this paper, Hamley et al.[6]
$ATP(t) = ABR \times L3(t)$	annual transmission potential	defined by ABR and $L3(t)$	Basáñez and Boussinesq[4]
$\Pi_{H(i)}\left[ATP(t-\tau_H), \Omega_T(a_{(i)}-\tau_H)\right] = \left[\frac{\delta_{H0}+\delta_{H\infty}c_H ATP(t-\tau_H)\Omega_T(a_{(i)}-\tau_H)}{1+c_H ATP(t-\tau_H)\Omega_T(a_{(i)}-\tau_H)}\right]$	density-dependent proportion of infective L3 larvae successfully establishing as adult worms	defined by $ATP(t-\tau_H)$<br>δ_{H0}, $\delta_{H\infty}$, c_H and $\Omega_T(a_{(i)}-\tau_H)$	Basáñez and Boussinesq[4]<br>Basáñez et al.[7]<br>Hamley et al.[6]
δ_{H0}	proportion of L3 larvae developing to the adult stage within the human host, per bite, when $ATP(t) \to 0$	0.19	Hamley et al.[6]
$\delta_{H\infty}$	proportion of L3 larvae developing to the adult stage within the human host, per bite, when $ATP(t) \to \infty$	2.7x10^{-3}	Hamley et al.[6]
c_H	severity of transmission intensity-dependent parasite establishment within humans	4.9x10^{-3}	Hamley et al.[6]
τ_H	time delay between L3 entering the human hosts and establishing as adult worms	0.8 years	Prost[9]

Parasite demography

Parasite mortality**.** The mortality rate, $\mu_l(a)$, of both adult worms and microfilariae is assumed to increase as a function of parasite age, according to a Weibull distribution of survival times, $S_l(a)$,

$$\mu_l(a) = {y_l}^{d_l} d_l a^{d_l - 1} \quad (6)$$

$$S_l(a) = e^{-y_l a^{d_l}} \quad (7)$$

where l denotes the parasite life stage W (adult worms) or M (microfilariae). Parameters y_l and d_l were estimated by fitting equation (S7) to data from Karam et al.[10] for adult worms and to data from Duke[11] for microfilariae (see Hamley et al.).[6] The life expectancy of each life stage is given by $\overline{L_l} = \int_0^\infty S_l(a)\, da$.

Assuming a default duration of one year for each worm age class, a maximum number of discrete age classes $c_{\max}$, and a maximum life span for each life stage L_l, we adjusted each age class to the appropriate duration, q_l, where l is the parasite life stage. Worm mortality and movement between age classes is modelled, like other transitions, as a discrete-time stochastic process. Worms leave an age class either due to death or progression to the next age class following,

$$
\begin{aligned}
&W_{s(i,a)}(t) \to W_{s(i,a)}(t+dt) - 1 && \text{with probability: } \left(\mu_W(a) + \frac{1}{q_W}\right) W_{s(i,a)}dt \\
& && \text{for } a \le L_W \\
&W_{s(i,a+q_W)}(t) \to W_{s(i,a+q_W)}(t+dt) + 1 && \text{with probability: } \frac{W_{s(i,a)}}{q_W}dt \\
& && \text{for } 0 < a < L_W
\end{aligned}
\tag{8}
$$

<u>Parasite fecundity and fertility</u>**.** Newly established adult female *O. volvulus*, are initially non-fertile, $W_{FN(i,a)}$, and progress to become fertile, $W_{FF(i,a)}$,[12] at a per capita rate ω.[12] Female worms only produce microfilariae when they are in the fertile state and in the presence of at least one co-infecting male worm (assuming complete polygamy of male worms such that one male can mate with all females within the same human host).[13,14] The stochastic process governing the rate of progression to fertility is defined as,

$$
\begin{aligned}
&W_{FN(i,a)}(t) \to W_{FN(i,a)}(t+dt) - 1 \\
&W_{FF(i,a)}(t) \to W_{FF(i,a)}(t+dt) + 1 && \text{with probability: } \omega W_{FN(i,a)}dt
\end{aligned}
\tag{9}
$$

Because female worms need to be re-inseminated at each reproductive cycle,[13,15] we further assume that fertile female worms lose their fertility and return to the non-fertile state at the per capita rate λ_0 such that,

$$
\begin{aligned}
&W_{FN(i,a)}(t) \to W_{FN(i,a)}(t+dt) - 1 \\
&W_{FF(i,a)}(t) \to W_{FF(i,a)}(t+dt) + 1 && \text{with probability: } \lambda_0 W_{FF(i,a)}dt
\end{aligned}
\tag{10}
$$

The contribution of each age class of adult female worms to the number of microfilariae in individual i depends on the fecundity rate, $m(a)$, and on the presence of at least one male (*M*) worm,

$$
\begin{aligned}
&m(a) = \frac{\varepsilon^* F}{F + G^{-a} - 1} && \text{for } \sum_{a=0}^{a=L_W} W_{M(i,a)}(t) > 0 \\
&m(a) = 0 && \text{for } \sum_{a=0}^{a=L_W} W_{M(i,a)}(t) = 0
\end{aligned}
\tag{11}
$$

where ε^* is the fecundity rate at age 0, and F and G determine the decline in fecundity with worm age. Supplementary Equation 11 was parameterized to mimic, with a nonlinear function[6], the modelled age-dependent fecundity used by Plaisier et al.,[16] which assumes that female worms have maximum fecundity during the first 5 years of age followed by a linear reduction of fecundity which becomes zero at the age of 21 years.

Microfilarial population dynamics. The change in the density of microfilariae per mg of skin is calculated deterministically by the partial differential equation,

$$\frac{\partial M_{(i)}}{dt} + \frac{\partial M_{(i)}}{da} = -\mu_M(a)M_{(i)}(a,t) \tag{12}$$

where $M_{(i)}(0,t) = \sum_{a=0}^{a=L_W} W_{FF(i,a)}(t)m(a)$ is the sum of the reproductive output of all adult female worms within a host. Note that computationally, we discretise microfilarial age classes in the same way as adult worms (depending on $c_{\max}$, L_M, q_M), but calculate changes in the microfilarial density by age and time by numerical integration. All parameters and variables in EPIONCHO-IBM that describe parasite demography are summarised in Supplementary

Supplementary **Table 5**.

Supplementary Table 5. EPIONCHO-IBM parameter definitions for parasite demography

Parameter	Definition	Value	Reference
y_W	parameter relating mortality rate to age in adult worms	0.1	Hamley et al.[6]
d_W	parameter relating mortality rate to age in adult worms	6.01	Hamley et al.[6]
y_M	parameter relating mortality rate to age in microfilariae	1.09	Hamley et al.[6]
d_M	parameter relating mortality rate to age in microfilariae	1.43	Hamley et al.[6]
L_W	maximum longevity of adult worms	20 years	Plaisier et al.[16]
L_M	maximum longevity of microfilariae	2.5 years	Duke[11]
$c_{\max}$	number of discrete age classes in both parasite life stages	21	Hamley et al.[6]
q_M	duration of each age class for microfilariae	0.125 years	Hamley et al.[6]
q_W	duration of each age class for adult worms	1 year	Hamley et al.[6]

ε^*	per capita rate of production of microfilariae per mg of skin per (fertile) adult female *Onchocerca volvulus* at age zero	1.15 year^{-1}	Basáñez et al.[12]
ω	per capita rate of progression from non-fertile to fertile adult female *O. volvulus* in Supplementary Equation 9	0.59 year^{-1}	Basáñez et al.[1,12]
λ_0	per capita rate of reversion from fertile to non-fertile adult female *O. volvulus* in Supplementary Equation 10	0.33 year^{-1}	Basáñez et al.[1,12]
F	parameter relating parasite fecundity to age	70	Hamley et al.[6]
G	parameter relating parasite fecundity to age	0.72	Hamley et al.[6]

***Onchocerca volvulus* larvae and vector population**

The vector transmission cycle is modelled deterministically. An individual's contribution to the larval burden in the vector population (subscript V) is calculated as a function of their microfilarial load. This contribution depends on the biting rate per fly on humans, and the individual's specific relative exposure to blackfly bites, $\Omega_T(a_{(i)})$. Much like the establishment of L3 larvae in the human host, the establishment of microfilariae in the simuliid vector is determined by a constraining density-dependent function. This density dependence is defined by $\Pi_{V(i)}(t)$ (Supplementary Supplementary Table **6**), such that the proportion of microfilariae developing into infective L3 larvae within the blackfly vector declines with increasing number of microfilariae ingested.[1,4] Additionally, this proportion is affected by the probability that a blackfly survives the extrinsic incubation period (EIP), the time it takes for ingested microfilariae to develop into L3 larvae (which represents a substantial proportion of the blackfly's lifespan). The EIP is incorporated by modelling explicitly the L1, L2 and L3 stages of development within the blackfly vector[2,17] whilst also accounting for a delay (τ_V) before L1 can start transitioning to the L2 stage, in line with experimental blackfly infection data.[18] The within-blackfly dynamics of L1, L2 and L3 stages are defined by,

$$\frac{dL1_{(i)}(t)}{dt} = \beta\Pi_{V(i)}(t)\Omega_T(a_{(i)}).M_{(i)}(t) - L1_{(i)}(t)[\mu_V + \alpha_V M_{(i)}(t)\Omega_T(a_{(i)})] - L1_{(i)}(t-\tau_V)\,\nu_1\,e^{-\tau_V[\mu_V + \alpha_V M_{(i)}(t-\tau_V)\Omega_{T(i)}(a_{(i)}-\tau_V)]} \quad (13)$$

$$\frac{dL2_{(i)}(t)}{dt} = L1_{(i)}(t-\tau_V)\,\nu_1\,e^{-\tau_V[\mu_V + \alpha_V M_{(i)}(t-\tau_V)\Omega_T(a_{(i)}-\tau_V)]} - L2_{(i)}(t)(\nu_2 + \mu_V) \quad (14)$$

$$\frac{dL3_{(i)}(t)}{dt} = \nu_2 L2_{(i)}(t) - L3_{(i)}(t)\left(\mu_V + \mu_{L3} + \left(\frac{a_H}{g}\right)\right) \quad (15)$$

Here, L1 larvae develop into L2 larvae at per capita rate ν_1 (estimated from *Simulum damnosum* s.l. data from Cameroon)[19] if they survive for at least time τ_V, or are lost when a blackfly vector dies. The

life expectancy of vectors is dependent on the number of microfilariae ingested per bite, the third constraining density-dependent process modelled in the parasite life-cycle. This parasite-induced excess mortality of blackflies is included as the product of a per microfilaria excess mortality term, α_V multiplied by the number of microfilariae ingested $M_{(i)}$.[1,4,18] Infective L3 larvae develop from L2 (pre-infective larvae) at per capita rate ν_2 (estimated from data in Eichner et al.)[19] and are lost from vectors as a result of three possible processes, namely, at per capita mortality rate μ_{L3}, when a blackfly dies at per capita rate μ_V, or upon inoculation into a vertebrate host (including humans) when a blackfly bites at rate a_H/g, i.e., the product of the proportion of L3 shed per bite (on any blood host), a_H, and the per capita rate of biting $1/g$, where g is the length of the gonotrophic cycle.[1,4] The mean number of L3 per blackfly is denoted $L3$. This provides the dynamic input to the ATP expression which defines the human hosts' annual rate of acquisition of new parasites in Supplementary Equation 5. The parameters defining the dynamics for the vector stages of *O. volvulus* larvae and the adult female fly population are given in Supplementary Supplementary Table **6**.

Supplementary Table 6. EPIONCHO-IBM parameter definitions for larval stages within the vector and adult female blackfly population dynamics

Parameter or variable	Definition	Value	Reference
$\Pi_{V(i)}(t) = \frac{\delta_{V0}}{[1 + c_V M_{(i)}(t)\Omega_T(a_{(i)})]}$	proportion of microfilariae (mf) per mg of skin in human host i developing into infective L3 larvae within the blackfly vector per bite	defined by δ_{V0}, c_V and $M_{(i)}(t)$	Basáñez and Boussinesq[4]
δ_{V0}	proportion of mf per mg developing to the infective L3 stage per bite when $M_{(i)}(t) \to 0$	0.0207	Basáñez and Boussinesq[4]
c_V	severity of density-dependent limitation of larval development per dermal microfilaria	0.00878	Basáñez and Boussinesq[4]
ν_1	per capita development rate from L1 to L2 larvae	201.6 year^{-1}	Eichner[19] Cheke et al.[20]
ν_2	per capita development rate from L2 to L3 larvae	207.7 year^{-1}	Eichner[19] Cheke et al.[20]
μ_V	per capita mortality rate of blackfly vectors	26 year^{-1}	Basáñez and Boussinesq[4]
α_V	per capita excess rate mortality on blackfly vectors induced by mf	0.39 year^{-1}	Basáñez and Boussinesq[4]
μ_L	per capita mortality rate of L3 larvae within blackfly vectors	52 year^{-1}	Basáñez and Boussinesq[4]
a_H	proportion of L3 larvae shed per bite	0.8	Basáñez and Boussinesq[4]

τ_V	delay before L1 larvae can start transitioning to L2 stage	0.011 years (4 days)	Eichner[19] Cheke et al.[20]

Single treatment with ivermectin

The pharmacodynamics of skin microfilarial load and the proportion of adult female worms producing live mf following ivermectin treatment (with the standard dose of 150 mg/kg dose) are modelled according to parameterization presented in Basáñez et al.[12] The microfilaricidal effect is modelled as a per capita excess mortality rate $\mu'_M(\tau_{(i)})$ that depends on the time elapsed since treatment, τ. Hence, following treatment with ivermectin, Supplementary Equation 12 is modified to,

$$\frac{\partial M_{(i)}}{dt} + \frac{\partial M_{(i)}}{da} = -\left(\mu_M(a) + \mu'_M(\tau_{(i)})\right) M_{(i)}(a,t) \quad (16)$$

where $\mu'_M(\tau_{(i)}) = (\tau_{(i)} + u)^{-\kappa}$. This function permits a very large but finite microfilaricidal effect at the point of treatment, defined by u, followed by a decline with shape governed by κ. The temporary sterilisation of adult female worms (the so-called embryostatic effect) following treatment with ivermectin is modelled by including a treatment-induced excess per capita rate $\lambda'(\tau_{(i)})$ at which fertile females become non-fertile. Hence, female worms move between the fertile and non-fertile compartments at a rate,

$$\begin{aligned} &W_{FN(i,a)}(t) \to W_{FN(i,a)}(t+dt) + 1, \\ &W_{FF\,(i,a)}(t) \to W_{FF\,(i,a)}(t+dt) - 1 \qquad \text{with probability: } [\lambda_0 + \lambda'(\tau_{(i)})]W_{FF\,(i,a)}dt \end{aligned} \quad (17)$$

Here, $\lambda'(\tau_{(i)}) = \lambda^{max} e^{(-\varphi\tau_{(i)})}$, where λ^{max} is the maximum rate of treatment-induced sterility and φ is the rate of decay of this effect with time after treatment. Supplementary Supplementary Table **7** provides definitions and values of the parameters that determine the parasite dynamics following ivermectin administration.

Supplementary Table 7. EPIONCHO-IBM parameter definitions for the anti-parasitic effects of a single treatment with ivermectin.

Parameter or variable	Definition	Value	Reference
$\mu'_M(\tau_{(i)}) = (\tau_{(i)} + u)^{-\kappa}$	ivermectin-induced per capita rate of excess mortality of microfilariae at time τ since treatment	defined by u and κ	Basáñez et al.[12]

u	constant to allow for very large yet finite microfilaricidal effect upon treatment with ivermectin	9.6×10^{-3}	Basáñez et al.[12]
κ	shape parameter for excess mortality of mf following treatment with ivermectin	1.25	Basáñez et al.[12]
$\lambda'(\tau_{(i)}) = \lambda^{max} e^{(-\varphi\tau_{(i)})}$	ivermectin-induced per capita rate of reversion from fertile to non-fertile adult female parasites at time τ since the last treatment	defined by λ^{max} and φ	Basáñez et al.[12]
λ^{max}	maximum rate of ivermectin-induced sterility	32.4 year^{-1}	Basáñez, et al.[12]
φ	rate of decay of ivermectin induced sterilisation	19.6 year^{-1}	Basáñez, et al.[12]

Treatment with a hypothetical macrofilaricide

The pharmacodynamics of a hypothetical macrofilaricidal drug are modelled by adding an excess mortality term $\mu'_W(\tau_{(i)})$ to the adult worm (male and female) per capita mortality rate following treatment. This function is parameterized to ensure that an assumed fraction (the macrofilaricidal efficacy, ϵ) of worms (of any age) is killed over a finite time period, τ^*,

$$\mu'_W(\tau_{(i)}) = \frac{\ln(1-\epsilon)}{\tau^*} dt \qquad \text{for } \tau_{(i)} \leq \tau^* \text{ and 0 otherwise.} \tag{18}$$

Thus, following treatment, the mortality rate of adult worms is given by adding this excess mortality to the (background) per capita mortality rate $\mu_l(a)$ given in Supplementary Equation 6,

$$\mu_W(a) = y_W{}^{d_W} d_W a^{d_W-1} + \mu'_W(\tau_{(i)}). \tag{19}$$

Parameters defining the modelled macrofilaricidal effects are defined in Supplementary Supplementary Table **8**.

Supplementary Table 8. EPIONCHO-IBM parameter definitions for the anti-parasitic effects of treatment with a hypothetical macrofilaricide.

Parameter	Definition	Value	Reference
$\mu'_W(\tau_{(i)}) = \frac{\ln(1-\epsilon)}{\tau^*} dt$ for $\tau_{(i)} \leq \tau^*$ and 0 otherwise	per capita excess mortality of adult *Onchocerca volvulus* induced by treatment with hypothetical macrofilaricide	defined by ϵ and τ^*	this paper
ϵ	proportion of adult *O. volvulus* killed by hypothetical macrofilaricide (efficacy)	varied; $\epsilon = 0.6$, $\epsilon = 0.75$, or $\epsilon = 0.9$	this paper
τ^*	time period over which *O. volvulus* worms are killed by treatment	0.25 years	this paper

Infection intensity and prevalence measures

Microfilariae are assumed to be aggregated within the skin[21,22] such that that the *observed* number of mf, $M^{*}_{(i)}(t)$, in a single skin snip of weight w is a random variable following a negative binomial distribution with (modelled) mean $M_{(i)}(t)w$ and overdispersion (aggregation) parameter $k_{M(i)}$,

$$M^{*}_{(i)}(t) \sim \mathrm{NBD}\big(M_{(i)}(t)w, k_{M(i)}\big) \quad (20)$$

The degree of aggregation of skin mf is assumed to *decrease* with increasing numbers of adult female worms and is parametrized as,

$$k_{M(i)} = 0.0478 \times W_{F(i)} + 0.313 \quad (21)$$

using estimates of the overdispersion parameter and the mean number of adult female worms per host reported in Bottomley et al.[22] This sampling model of mf has been validated in Hamley et al.[6] against independent repeated data on 40 repeat skin snips (microfilarial counts) taken from 15 individuals over 24 hours as reported in Picq and Jardel.[23] Typically, n skin snips (in this paper $n = 2$ or $n = 4$) are taken from each host to estimate the microfilarial density per milligram of skin, $\bar{M}^{*}_{(i)}(t)$. Hence, for $k = 1 \ldots n$ skin snips of weight w,

$$\bar{M}^{*}_{(i)}(t) = \frac{1}{nw}\sum_{k=1}^{k=n} M^{*}_{(i,k)}(t). \quad (22)$$

The mean number of mf per mg of skin per human host is,

$$\bar{M}^{*}(t) = \frac{1}{N}\sum_{i=1}^{i=N} \bar{M}^{*}_{(i)}(t). \quad (23)$$

Prevalence is calculated in the same manner, converting $\bar{M}^{*}_{(i)}$to a binary variable such that,

$$\bar{P}^{*}_{(i)}(t) = 1\ for\ \bar{M}^{*}_{(i)}(t) > 0 \text{ and 0 otherwise} \quad (24)$$

and

$$\bar{P}^{*}(t) = \frac{1}{N}\sum_{i=1}^{i=N} \bar{P}^{*}_{(i)}(t). \quad (25)$$

Parameters defining the measures of parasite intensity and prevalence used in this paper are given in

Supplementary Table 9.

Supplementary Table 9. EPIONCHO-IBM parameter definitions for infection intensity and prevalence measures.

Parameter or variable	Definition	Value	Reference

$M^{*}_{(i)}(t)$	the observed number of microfilariae (mf) in a single skin snip of weight w from human host i	model output	Hamley et al.[6]
w	the weight of skin in one skin snip	2 mg	Awadzi et al.[24]
$\bar{M}^{*}_{(i)}(t)$	the number of mf per mg skin from n skin snips in human host i	model output	Hamley et al.[6]
$\bar{P}^{*}_{(i)}(t)$	the proportion of n skin snips positive for mf in human host i	model output	Hamley et al.[6]
n	the number of skin snips taken per individual human host	varied; $n = 2$ or $n = 4$	this paper
$\bar{M}^{*}(t)$	the mean number of mf per mg of skin per human host	model output	Hamley et al.[6]
$k_{M(i)} = k_{M0} + k_{M1}W_{F(i)}$	the degree of aggregation of microfilariae (mf) within the skin of human host i	defined by k_{M0} and k_{M1}	Hamley et al.[6]
k_{M0}	the degree of aggregation of mf in the skin of human hosts as $W_{F(i)}(t) \to 0$	0.313	Hamley et al.[6]
k_{M1}	the rate of change in aggregation with increasing $W_{F(i)}(t)$	0.0478 per adult female worm	Hamley et al.[6]

Clinical trial outcome measures

The intensity (mf per mg and skin) and prevalence (percent positive for mf) are the two parasitological outcome measures simulated for evaluation of the clinical trial. These are calculated for sub-groups of participants meeting the trial eligibility criteria (aged ≥ 18 years; not pregnant or breastfeeding, either positive for mf (> 0) or with > 4 or > 8 mf per mg skin) just before administration of treatment. We use the prime notation to denote outcome measures from participating individuals and the $\tau_{(i)}$ time variable to indicate that these measures change with time after treatment (as opposed to time t since the start of the simulation). Importantly, the denominator of the trial outcome measures will also vary with $\tau_{(i)}$ since individuals either dying or lost to follow up are not replaced in the trial cohort. Hence, we denote $N_T(\tau)$ and $N_C(\tau)$ as the number of participants in the macrofilaricide-treated test and ivermectin-treated control cohorts, respectively, at time $\tau_{(i)}$ after treatment, where the target for $N_T(0) = N_C(0) = 50$ (i.e. a target of 50 participants initially selected for each cohort; note that this target number becomes increasingly difficult to meet with increasing microfilarial intensity inclusion criterion, Supplementary **Supplementary Figure 4**) for all simulations.

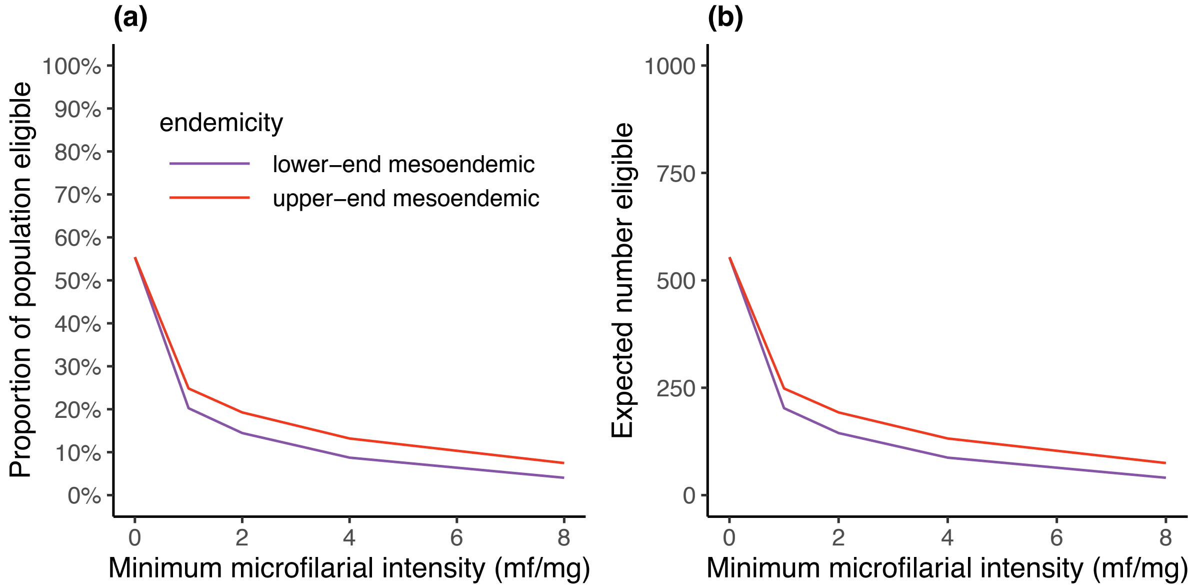

Supplementary Figure 4. The relationship between the expected number of individuals eligible for participation in a clinical trial with increasing microfilarial intensity inclusion criterion.

Panels (a) and (b) depict, respectively, the proportion and expected number of a population of 1,000 individuals meeting the inclusion criteria for participation in a clinical trial. Inclusion criteria are: age ≥ 18 years; not pregnant or breastfeeding, either positive for mf (minimum microfilarial intensity > 0 microfilariae, mf per mg skin) or greater than the value indicated on the *x*-axis.

Sample size calculations

We use $\widehat{D}(\tau)$ to denote generically the mean difference between measures of either intensity (mf per mg of skin) or prevalence (percent positive for mf) in the macrofilaricide-treated test and ivermectin-treated control groups from each simulation. That is, $\widehat{D}(\tau)$ is the mean response in the control group subtracted from the mean response in the macrofilaricide-treated test group for each of the 1,000 simulations run for each parameter combination (see Table 1 in the main text). We further denote $\hat{\sigma}_T(\tau)$ and $\hat{\sigma}_C(\tau)$ (T = test, C = control) as the standard deviation among responses from the 50-participant test and control cohorts included in each simulation. By invoking the central limit theorem, we assume that the distribution of $\widehat{D}(\tau)$ is approximately Gaussian such that Welch's *t* statistic,[25]

$$T(\tau) = \frac{\widehat{D}(\tau)}{\hat{\sigma}(\tau)} \tag{26}$$

follows a non-central *t*-distribution, where $\hat{\sigma}(\tau)$ is the approximate standard deviation among responses from both test and control groups and is estimated by,

$$\hat{\sigma}(\tau) = \sqrt{\frac{\hat{\sigma}_T^2(\tau)}{N_T(\tau)} + \frac{\hat{\sigma}_C^2(\tau)}{N_C(\tau)}}. \tag{27}$$

The degrees of freedom ν associated with $T(\tau)$ is approximated by the Welch-Satterthwaite equation,

$$\nu \approx \hat{\sigma}_T^4(\tau)\left(\frac{\hat{\sigma}_T^4(\tau)}{N_T^2(\tau)\nu_T}+\frac{\hat{\sigma}_C^4(\tau)}{N_C^2(\tau)\nu_C}\right)^{-1}, \tag{28}$$

where $\nu_T = N_T(\tau) - 1$ and $\nu_C = N_C(\tau) - 1$. We confirmed that the distribution of $T(\tau)$ was adequately approximated by replacing $\widehat{D}(\tau)$, $\hat{\sigma}_T(\tau)$, $\hat{\sigma}_C(\tau)$ with their 'true' values $D(\tau)$, $\sigma_T(\tau)$ and $\sigma_C(\tau)$ approximated from the 1,000 repeated simulations. Hence, the probability (power, β) that $T(\tau)$ exceeds the critical value of the *t* distribution t_α, where $1 - \alpha = 0.05$ is the (one-sided) probability of rejecting the null hypothesis that $D(\tau) > 0$ (type I error), is given by,

$$\beta = 1 - G(t_\alpha), \tag{29}$$

where $G(t_\alpha)$ is the cumulative distribution function of the non-central *t*-distribution with non-centrality parameter $D(\tau)$ and degrees of freedom ν. Assuming equal starting cohort sizes $N(0) = N_T(0) = N_C(0)$ and 10% drop out per year, we used Supplementary Equation 29 to find the smallest integer $N(0)$ that ensured a power of at least 80%, $\beta = 0.8$.

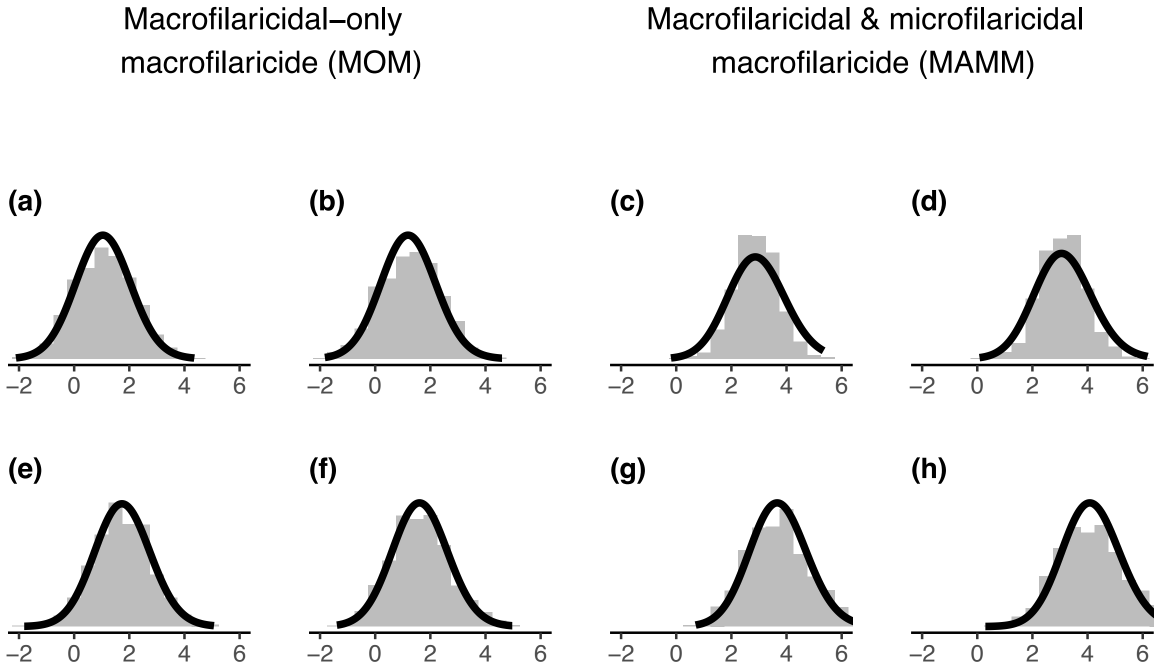

Supplementary Figure 5. Distribution of Welch's *t*-statistic generated from 1,000 clinical trial simulations using an inclusion criterion of > 0 microfilariae per mg.

Each panel depicts Welch's *t*-statistic generated from 1,000 clinical trial simulations (grey bars) and the predicted density function (solid black line) of the non-central *t*-distribution using the estimated 'true' values of the difference and standard deviation of responses in the macrofilaricide-treated test and ivermectin-treated control groups. Simulation results correspond to a 12-month follow up after treatment with a macrofilaricide of efficacy 90%. Panels **(a)** to **(d)** correspond to a microfilarial intensity outcome measure; panels **(e)** to **(h)** to a microfilarial prevalence outcome. Panels **(a)**, **(c)**, **(e)** and **(g)** correspond to simulations assuming 2 skin snips per participant; panels **(b)**, **(d)**, **(f)** and **(h)** to simulations assuming 4 skin snips per participant.

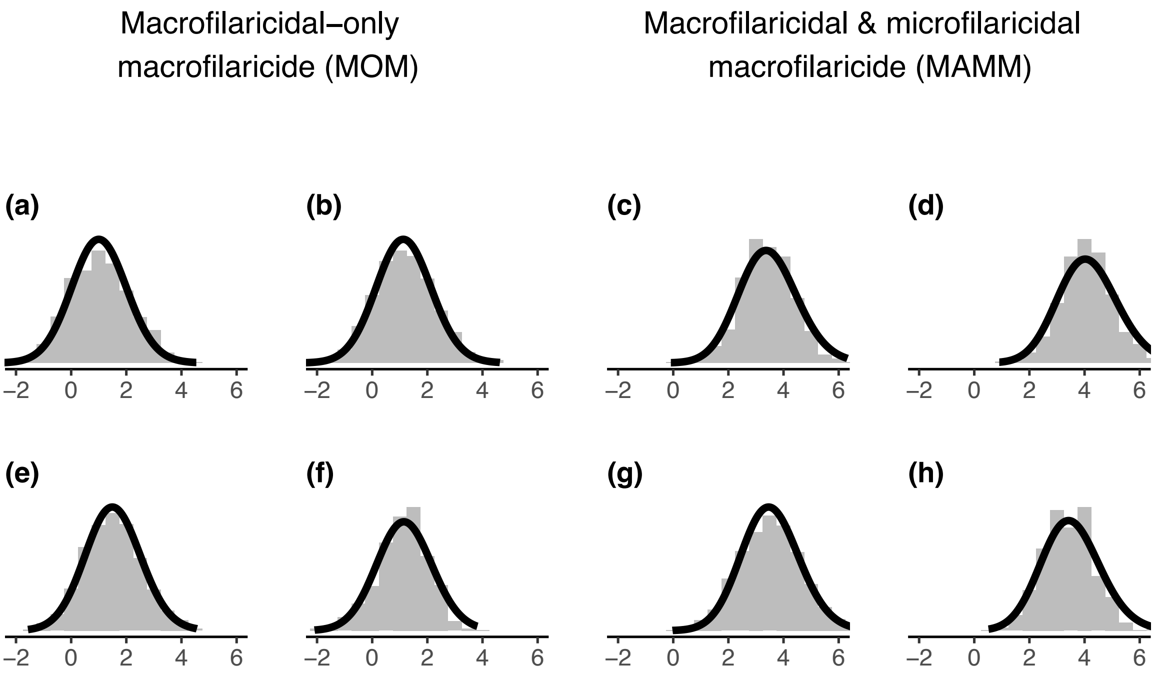

Supplementary Figure 6. Distribution of Welch's *t*-statistic generated from 1,000 clinical trial simulations using an inclusion criterion of > 4 microfilariae per mg

Each panel depicts Welch's *t*-statistic generated from 1,000 clinical trial simulations (grey bars) and the predicted density function (solid black line) of the non-central t-distribution using the estimated 'true' values of the difference and standard deviation of responses in the macrofilaricide-treated test and ivermectin-treated control groups. Simulation results correspond to a 12-month follow up after treatment with a macrofilaricide of efficacy 90%. Panels **(a)** to **(d)** correspond to a microfilarial intensity outcome measure; panels **(e)** to **(h)** to a microfilarial prevalence outcome. Panels **(a)**, **(c)**, **(e)** and **(g)** correspond to simulations assuming 2 skin snips per participant; panels **(b)**, **(d)**, **(f)** and **(h)** to simulations assuming 4 skin snips per participant.

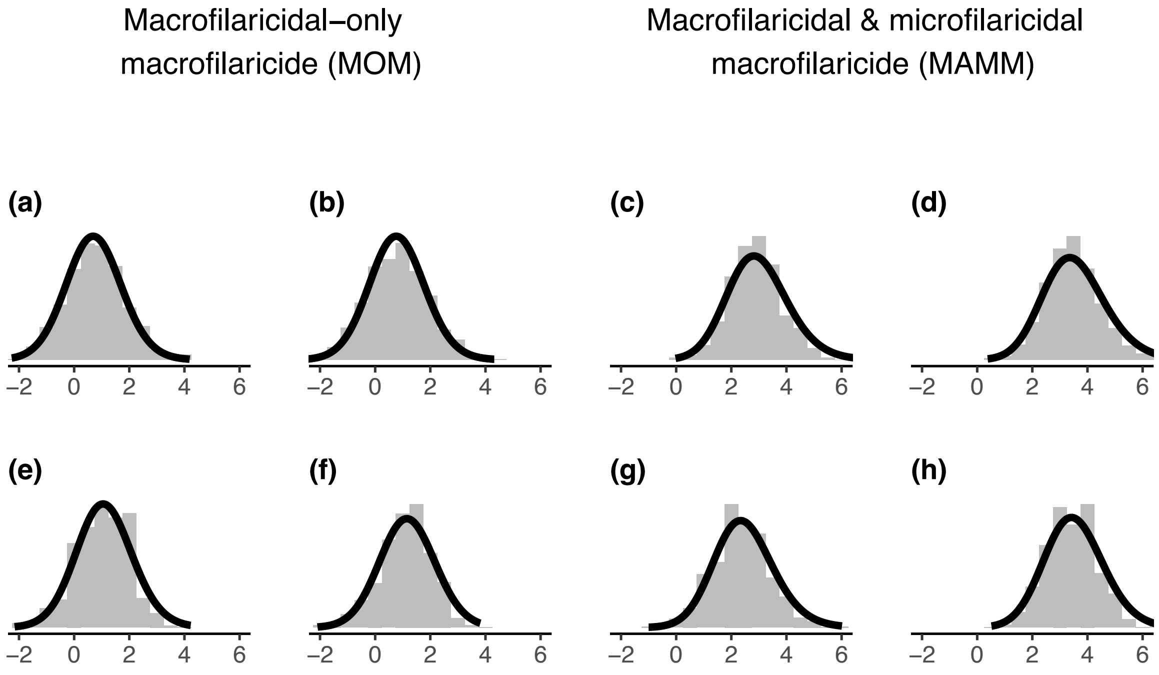

Supplementary Figure 7 Distribution of Welch's *t*-statistic generated from 1,000 clinical trial simulations using an inclusion criterion of > 8 microfilariae per mg.

Each panel depicts Welch's *t*-statistic generated from 1,000 clinical trial simulations (grey bars) and the predicted density function (solid black line) of the non-central t-distribution using the estimated 'true' values of the difference and standard deviation of responses in the macrofilaricide-treated test and ivermectin-treated control groups. Simulation results correspond to a 12-month follow up after treatment with a macrofilaricide of efficacy 90%. Panels **(a)** to **(d)** correspond to a microfilarial intensity outcome measure; panels **(e)** to **(h)** to a microfilarial prevalence outcome. Panels **(a)**, **(c)**, **(e)** and **(g)** correspond to simulations assuming 2 skin snips per participant; panels **(b)**, **(d)**, **(f)** and **(h)** to simulations assuming 4 skin snips per participant.

Supplementary References

1 Basáñez, M. G. *et al.* River blindness: Mathematical models for control and elimination. *Adv Parasitol* **94**, 247-341, doi:10.1016/bs.apar.2016.08.003 (2016).

2 Walker, M. *et al.* Modelling the elimination of river blindness using long-term epidemiological and programmatic data from Mali and Senegal. *Epidemics* **18**, 4-15, doi:10.1016/j.epidem.2017.02.005 (2017).

3 Prost, A. & Gorim de Ponsay, E. [The epidemiological significance of neo-natal parasitism with microfilariae of *Onchocerca volvulus* (author's transl)]. *Tropenmed Parasitol* **30**, 477-481 (1979). In French.

4 Basáñez, M. G. & Boussinesq, M. Population biology of human onchocerciasis. *Philos Trans R Soc Lond B Biol Sci* **354**, 809-826, doi:10.1098/rstb.1999.0433 (1999).

5 Filipe, J. A. N. *et al.* Human infection patterns and heterogeneous exposure in river blindness. *Proc Natl Acad Sci U S A* **102**, 15265-15270, doi:10.1073/pnas.0502659102 (2005).

6 Hamley, J. I. D., Milton, P., Walker, M. & Basáñez, M. G. Modelling exposure heterogeneity and density dependence in onchocerciasis using a novel individual-based transmission model, EPIONCHO-IBM: implications for elimination and data needs. *PLoS Negl Trop Dis* **13**, e0007557, doi:10.1371/journal.pntd.0007557 (2019).

7 Basáñez, M. G., Collins, R. C., Porter, C. H., Little, M. P. & Brandling-Bennett, D. Transmission intensity and the patterns of *Onchocerca volvulus* infection in human communities. *Am J Trop Med Hyg* **67**, 669-679 (2002).

8 Lamberton, P. H. L. *et al.* Onchocerciasis transmission in Ghana: the human blood index of sibling species of the *Simulium damnosum* complex. *Parasit Vectors* **9**, 432, doi:10.1186/s13071-016-1703-2 (2016).

9 Prost, A. [Latency period in onchocerciasis]. *Bull World Health Organ* **58**, 923-925 (1980). In French.

10 Karam, M., Schulz-Key, H. & Remme, J. Population dynamics of *Onchocerca volvulus* after 7 to 8 years of vector control in West Africa. *Acta Trop* **44**, 445-457 (1987).

11 Duke, B. O. L. The effects of drugs on *Onchocerca volvulus*. 1. Methods of assessment, population dynamics of the parasite and the effects of diethylcarbamazine. *Bull World Health Organ* **39**, 137-146 (1968).

12 Basáñez, M. G. *et al.* Effect of single-dose ivermectin on *Onchocerca volvulus*: a systematic review and meta-analysis. *Lancet Infect Dis* **8**, 310-322, doi:10.1016/S1473-3099(08)70099-9 (2008).

13 Schulz-Key, H. & Karam, M. Periodic reproduction of *Onchocerca volvulus*. *Parasitol Today* **2**, 284-286 (1986).

14 May, R. M. Togetherness among schistosomes: its effects on the dynamics of the infection. *Math Biosci* **35**, 301-343, doi:10.1016/0025-5564(77)90030-X (1977).

15 Schulz-Key, H. Observations on the reproductive biology of *Onchocerca volvulus*. *Acta Leiden* **59**, 27-44 (1990).

16 Plaisier, A. P., van Oortmarssen, G. J., Remme, J. & Habbema, J. D. The reproductive lifespan of *Onchocerca volvulus* in West African savanna. *Acta Trop* **48**, 271-284 (1991).

17 Basáñez, M. G., Razali, K., Renz, A. & Kelly, D. Density-dependent host choice by disease vectors: epidemiological implications of the ideal free distribution. *Trans R Soc Trop Med Hyg* **101**, 256-269, doi:10.1016/j.trstmh.2006.08.009 (2007).

18 Basáñez, M. G., Churcher, T. S. & Grillet, M. E. *Onchocerca-Simulium* interactions and the population and evolutionary biology of *Onchocerca volvulus*. *Adv Parasitol* **68**, 263-313, doi:10.1016/S0065-308X(08)00611-8 (2009).

19 Eichner, M. *Onchocerca volvulus (Nematoda, Filarioidea) und Simulium damnosum-Komplex (Diptera): Die Entwicklung intrathorakal injizierter Mikrofilarien in verschiedenen Überträgerspecies Kameruns.* Diplomarbeit thesis, Universität Tübingen (1989).

20 Cheke, R. A. *et al.* Potential effects of warmer worms and vectors on onchocerciasis transmission in West Africa. *Philos Trans R Soc Lond B Biol Sci* **370**, doi:10.1098/rstb.2013.0559 (2015).

21 Kershaw, W. E., Duke, B. O. L. & Budden, F. H. Distribution of microfilariae of *O. volvulus* in the skin; its relation to the skin changes and to eye lesions and blindness. *Br Med J* **2**, 724-729 (1954).

22 Bottomley, C. *et al.* Modelling Neglected Tropical Diseases diagnostics: the sensitivity of skin snips for *Onchocerca volvulus* in near elimination and surveillance settings. *Parasit Vectors* **9**, 343, doi:10.1186/s13071-016-1605-3 (2016).

23 Picq, J. J. & Jardel, J. P. [(A method of evaluating microfilaria densities of *Onchocerca volvulus* Leuckart, 1893, in onchoceriasis patients. Assessment of microfilarial densities by site and levels of prevelance in skin biopsies; variations of microfilarial densities over a 24 hour period)]. *Bull World Health Organ* **51**, 145-153 (1974). In French.

24 Awadzi, K., Opoku, N. O., Attah, S. K., Lazdins-Helds, J. & Kuesel, A. C. A randomized, single-ascending-dose, ivermectin-controlled, double-blind study of moxidectin in *Onchocerca volvulus* infection. *PLoS Negl Trop Dis* **8**, e2953, doi:10.1371/journal.pntd.0002953 (2014).

25 Welch, B. L. The generalisation of student's problems when several different population variances are involved. *Biometrika* **34**, 28-35 (1947).
