# Supplementary material for: Designing antifilarial drug trials using clinical trial simulators
Source: Nat Commun. 2020 Jun 1;11:2685. doi: 10.1038/s41467-020-16442-y (PMC7264235; doi:10.1038/s41467-020-16442-y)
Supplement: Supplementary file 3 — Reporting Summary [file 41467_2020_16442_MOESM3_ESM.pdf]

nature research

Corresponding author(s): Martin Walker

Last updated by author(s): Apr 29, 2020

Reporting Summary

Nature Research wishes to improve the reproducibility of the work that we publish. This form provides structure for consistency and transparency in reporting. For further information on Nature Research policies, see Authors & Referees and the Editorial Policy Checklist .

Statistics

For all statistical analyses, confirm that the following items are present in the figure legend, table legend, main text, or Methods section.

n/a	Confirmed	
☐	☒	The exact sample size (n) for each experimental group/condition, given as a discrete number and unit of measurement
☐	☐	A statement on whether measurements were taken from distinct samples or whether the same sample was measured repeatedly
☐	☒	The statistical test(s) used AND whether they are one- or two-sided<br>*Only common tests should be described solely by name; describe more complex techniques in the Methods section.*
☒	☐	A description of all covariates tested
☒	☐	A description of any assumptions or corrections, such as tests of normality and adjustment for multiple comparisons
☐	☒	A full description of the statistical parameters including central tendency (e.g. means) or other basic estimates (e.g. regression coefficient) AND variation (e.g. standard deviation) or associated estimates of uncertainty (e.g. confidence intervals)
☒	☐	For null hypothesis testing, the test statistic (e.g. F, t, r) with confidence intervals, effect sizes, degrees of freedom and P value noted<br>*Give P values as exact values whenever suitable.*
☒	☐	For Bayesian analysis, information on the choice of priors and Markov chain Monte Carlo settings
☒	☐	For hierarchical and complex designs, identification of the appropriate level for tests and full reporting of outcomes
☒	☐	Estimates of effect sizes (e.g. Cohen's d, Pearson's r), indicating how they were calculated

Our web collection on statistics for biologists contains articles on many of the points above.

Software and code

Policy information about availability of computer code

Data collection	No software was used for data collection.
Data analysis	The analysis was performed using a custom-built mathematical model describing the transmission dynamics of Onchocerca volvulus. The model code is freely available from a GitHub repository at https://github.com/mrc-ide/EPIONCHO-IBM-CTS. Instructions for installing and running the model in R (version 3.6.2) are available in the README.html which can be accessed at https://mrc-ide.github.io/EPIONCHO-IBM-CTS/README.html.

For manuscripts utilizing custom algorithms or software that are central to the research but not yet described in published literature, software must be made available to editors/reviewers. We strongly encourage code deposition in a community repository (e.g. GitHub). See the Nature Research guidelines for submitting code & software for further information.

Data

Policy information about availability of data

All manuscripts must include a data availability statement. This statement should provide the following information, where applicable:

- Accession codes, unique identifiers, or web links for publicly available datasets
- A list of figures that have associated raw data
- A description of any restrictions on data availability

No external data were used. The individual-level simulated data generated during this study are available from the corresponding author on reasonable request.

Field-specific reporting

Please select the one below that is the best fit for your research. If you are not sure, read the appropriate sections before making your selection.

Life sciences study design

All studies must disclose on these points even when the disclosure is negative.

Sample size	This study uses a mathematical transmission model to estimate sample sizes required to demonstrate superiority of a (hypothetical) novel macrofilaricidal therapy compared to ivermectin (standard treatment). Sample sizes are estimated using Welch's t-statistic using approximations (obtained by numerical simulation) of the mean and standard deviation of the difference in responses between participants treated with either a macrofilaricide or ivermectin. A detailed description of the approach is given in the Methods section of the manuscript.
Data exclusions	No external data were used for the analysis. All data generated from the simulation modelling were included in the analysis.
Replication	A subset of the 72,000 simulations was repeated to confirm that the estimated sample sizes reported in the manuscript were reproducible.
Randomization	In the simulations, participants were assigned randomly to either the macrofilaricide-treated of the ivermectin-treated groups.
Blinding	Blinding is not relevant to this simulation study.

Reporting for specific materials, systems and methods

We require information from authors about some types of materials, experimental systems and methods used in many studies. Here, indicate whether each material, system or method listed is relevant to your study. If you are not sure if a list item applies to your research, read the appropriate section before selecting a response.

Materials & experimental systems

n/a	Involved in the study
☒	☐ Antibodies
☒	☐ Eukaryotic cell lines
☒	☐ Palaeontology
☒	☐ Animals and other organisms
☒	☐ Human research participants
☒	☐ Clinical data

Methods

n/a	Involved in the study
☒	☐ ChIP-seq
☒	☐ Flow cytometry
☒	☐ MRI-based neuroimaging
